# A new molecular marker for species-specific identification of *Microsporum canis*

**DOI:** 10.1007/s42770-020-00340-y

**Published:** 2020-07-21

**Authors:** Anita Ciesielska, Paweł Stączek

**Affiliations:** Department of Microbial Genetics, Faculty of Biology and Environmental Protection, University of Łódź, Łódź, Poland

**Keywords:** *Microsporum canis*, PCR, Molecular identification, Molecular marker

## Abstract

Species identification of dermatophytes by conventional mycological methods based on macro- and microscopy analysis is time-consuming and has a lot of limitations such as slow fungal growth or low specificity. Thus, there is a need for the development of molecular methods that would provide reliable and prompt identification of this group of medically important fungi. The are many reports in the literature concerning PCR identification of dermatophyte species, but still, there are not many PCR assays for the separate detection of members of the genera *Microsporum*, especially *Microsporum canis* (zoophilic species) and *Microsporum audouinii* (anthropophilic species). The correct distinction of these species is important to determine the source of infection to implement the appropriate action to eliminate the path of infection transmission. In this paper, we present such a PCR-based method targeting *velB* gene that uses a set of two primers—Mc-VelB-F (5′-CTTCCCCACCCGCAACATC-3′) and Mc-VelB-R (5′-TGTGGCTGCACCTGAGAGTGG-3′)*.* The amplified fragment is specific due to the presence of (CAGCAC)_8_ microsatellite sequence only in the *velB* gene of *M. canis.* DNA from 153 fungal samples was used in PCR assay followed by electrophoretic analysis. The specificity of the designed set of primers was also confirmed using the online BLAST-Primer tool. The positive results were observed only in the case of *M. canis* isolates, and no positive results were obtained neither for other dermatophytes and non-dermatophyte fungi nor for other Eukaryotes, including the human genome sequence, as well as the representatives of bacterial and viral taxa. The developed PCR assay using the proposed Mc-VelB-F and Mc-velB-R primers can be included in the algorithm of *M. canis* detection in animals and humans.

## Introduction

*Tinea corporis* and *tinea capitis* are the most common types of dermatophyte infections caused by *Microsporum canis*, less often by *M. audouinii* [1]. This kind of dermatophytosis occurs in all geographical areas, causing local endemics and even outbreaks, e.g., in kindergartens and schools. Traditional mycological identification mainly relied on a macroscopic and microscopic examination requiring experienced personnel [2]. Misidentification using conventional assays was the main reason for the development of molecular methods based mainly on the PCR technique, which became fast and reliable alternatives. Correct identification of *M. canis* as an etiological agent of infection and effective elimination of the source of the fungus are the factors necessary to prevent further transmission. There are only a few *M. canis* molecular identification assays described in the literature, in which the ITS1 sequence of the ribosomal DNA or β-tubulin gene was used as molecular markers [3–5]. Here, we present a new, alternative set of PCR primers—Mc-VelB-F and Mc-VelB-R—that are specific for the *velB* gene. The VelB (velvet-like B) protein is a light-dependent regulator that belongs to the velvet family proteins, which plays a key role in coordinating secondary metabolism and fungal development [6]. The purpose of this study was to validate the use of *velB* gene as a new molecular marker for species identification of *M. canis* isolated from humans and animals as well as differentiation of *M. canis* from *M. audouinii*.

## Material and methods

We used 153 DNA samples isolated from fungi belonging to genera *Trichophyton*, *Microsporum*, *Epidermophyton*, *Chrysosporium,* and *Candida* which were obtained from Westerdijk Fungal Biodiversity Institute collection (formerly CBS-KNAW, Centraalbureau voor Schimmelcultures, Utrecht, Netherlands) or originated from the collection maintained in the Department of Microbial Genetics (DMG), Faculty of Biology and Environmental Protection, University of Łódź, Poland (Table 1). Traditional mycological identification was confirmed by PCR-RFLP targeting the ITS1-5.8S-ITS2 region [7] and sequencing of PCR products. Based on the alignment (Geneious software) of fungal *velB* gene sequence deposited in the NCBI nucleotide database (Accession no. XM_002845600), primers specific for *M. canis*—Mc-VelB-F (5′-CTTCCCCACCCGCAACATC-3′) and Mc-VelB-R (5′-TGTGGCTGCACCTGAGAGTGG-3′)—were designed using Primer 3 software [8]. Each PCR mixture (20 μl) contained 1 μl of genomic DNA (20 ng), 0.5 μl of 0.5 μM of each primer, 4 μl 5xGC buffer, 0.4 μl 10 mM dNTPs, 1 μl DMSO, 12.4 μl distilled water, and 0.2 μl Phusion Hot Start II DNA Polymerase (Thermo Scientific). Reaction mixtures were preheated to 98 °C for 5 min, and then 35 PCR cycles were performed under the following conditions: 98 °C for 1 min, 68 °C for 1 min, and 72 °C for 1 min. Thermal cycles were finalized by polymerization at 72 °C for 5 min. The PCR products were purified with PCR Purification Kit (Qiagen), and detection was performed by electrophoresis in 12% polyacrylamide gel stained with ethidium bromide and visualized by UV light. The primer pair was tested for specificity using the online Primer-BLAST tool (https://www.ncbi.nlm.nih.gov/tools/primer-blast/). The primers were blasted against several nucleotide collection BLAST databases, including fungi (taxid: 4751), eukaryota (taxid: 2759), bacteria (taxid: 2), viruses (taxid: 10239), and human DNA (taxid: 9606). Default settings were used, except for the primer specificity stringency that was set to ignore targets that have six or more mismatches to the primer. Additionally, a BLAST (Basic Local Alignment Search Tool) search using available NCBI database sequences of the *velB* gene of *M. canis* and other dermatophytes as well as fungi belonging to *Ascomycota* was conducted to identify microsatellite motif (CAGCAC)_8._

**Table 1:** DNA samples used in the study and PCR results

Fungi species	Collection	No. of DNA samples	*velB*-specific PCR product (~ 200 bp)
*Microsporum canis* (A, Z)	CBS 113480, DMG	14 (A) 29 (Z)	Positive
*M. audouinii* (A)	CBS 102894	1	Negative
*Nannizzia gypsea* (G)	CBS 130813	1	Negative
*Trichophyton rubrum* (A)	CBS 120358, DMG	21	Negative
*T. interdigitale* (A)	CBS 120357, DMG	17	Negative
*T. tonsurans* (A)	CBS 109034, DMG	3	Negative
*T. verrucosum* (Z)	CBS 102011, DMG	4	Negative
*T. ajelloi* (G)	CBS 119779, DMG	17	Negative
*Epidermophyton floccosum* (A)	CBS 100148, DMG	4	Negative
*Chrysosporium keratinophilum* (Z)	CBS 104.62, DMG	21	Negative
*Ch. tropicum* (Z)	CBS 171.62, DMG	19	Negative
*Candida albicans*	DMG	2	Negative

A, anthropophilic; Z, zoophilic; G, geophilic; CBS, Centraalbureau voor Schimmelcultures (Westerdijk Fungal Biodiversity Institute); DMG, Department of Microbial Genetics

## Results and discussion

**Fig. 1:**
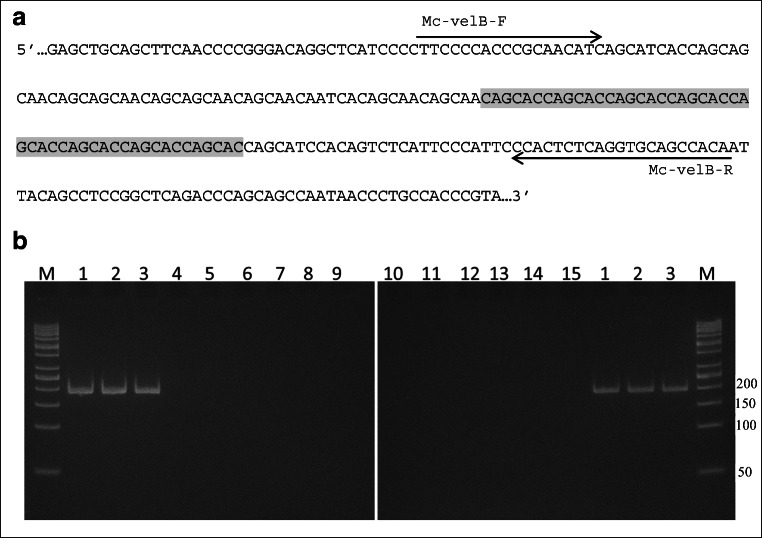
**a** Representation of a fragment of the target sequence of *velB* gene (Accession no. XM_002845600) and direction of primers. Shaded area represents the unique microsatellite motif (CAGCAC)_8_. **b** Exemplary specificity assay with the primers Mc-velB-F and Mc-velB-R. M, 50 bp DNA Ladder (Thermo Scientific). Lanes 1–15 PCR reactions using 20 ng of template DNA: 1, *Microsporum canis* CBS 113480; 2, *M. canis* DMG1; 3, *M. canis* DMG2; 4, *Nannizzia gypsea* CBS 130813; 5, *M. audouinii* CBS 102894; 6, *Trichophyton rubrum* CBS 120358; 7, *T. interdigitale* CBS 120357; 8, *T. tonsurans* CBS 109034; 9, *T. verrucosum* CBS 102011; 10, *T. ajjeloi* CBS 119779; 11, *Epidermophyton floccosum* CBS 100148; 12, *Chrysosporium keratinophilum* CBS 104.62; 13, *Ch. tropicum* CBS 171.62; 14, *C. albicans* DMG; 15, NTC, no template control. CBS, Westerdijk Fungal Biodiversity Institute culture collection; DMG, Department of Microbial Genetics collection

In silico PCR validation using an online Primer-BLAST search showed that the designed Mc-VelB-F and Mc-VelB-R primer set was binding exclusively to the target sequence (XM_002845600) of *Microsporum canis* CBS 113480 species. The results of this analysis revealed that the reported primer pair was very specific. The in vitro validation of these primers has shown that the PCR product of about 200 bp was detected only in the case of *M. canis* DNA samples (Table 1, Fig. 1b). It is worth to emphasize that the amplified fragment of *M. canis velB* gene is very unique because of the presence of the microsatellite motif (CAGCAC)_8_ (Fig. 1a). For that reason, we analyzed the available sequences of the *velB* gene in other dermatophytes as well as other fungi belonging to *Ascomycota* deposited in the NCBI database such as *Nannizzia gypsea* (XM_003175030), *Trichophyton rubrum* (XM_003232653), *Aspergillus niger* (XM_001389016), *Aspergillus flavus* (GU815258), *Aspergillus nidulans* (EF540815), *Aspergillus terreus* (KY425759), *Penicillium marneffei* (XM_002148380), *Ajellomyces dermatitidis* (XM_002625878), *Metarhizium brunneum* (XM_014688378), *Metarhizium acridum* (XM_007812729), *Verticillium dahliae* (XM_009651032), *Verticillium alfalfae* (XM_003004840), *Curvularia lunata* (KY435512), *Marssonina brunnea* (XM_007292607), *Beauveria bassiana* (JX156415), *Coccidioides immitis* (XM_001238939), *Colletotrichum orchidophilum* (XM_022618517), *Colletotrichum higginsianum* (XM_018295991), *Colletotrichum graminicola* (XM_008095148), *Pochonia chlamydosporia* (XM_018291621), *Gaeumannomyces tritici* (XM_009219313), *Magnaporthe oryzae* (XM_003714558), *Rasamsonia emersonii* (XM_013468828), *Talaromyces stipitatus* (XM_002482648), and *Beauveria bassiana* (XM_008604835). According to the bioinformatic analysis, we have noticed that the specific microsatellite motif (CAGCAC)_8_ located within the *velB* gene is present only in the case of *M. canis*. Computer analysis indicated also that the shorter (CAGCAC)_5_ motif was present in the case of *N. gypsea velB* gene. The CAGCAC sequence was present as a separate single motif in three locations within *velB* gene of *C. higginsianum*, twice in the case of *C. orchidophilum* and *B. bassiana,* and once in the case of *V. dahliae*, *V. alfalfae*, *M. acridum*, *C. lunata*, *A. nidulans*, *M. brunnea*, *B. bassiana* (JX156415), *P. chlamydosporia*, *M. oryzae,* and *T. stipitatus*. In the remaining analyzed species, no CAGCAC motif was found.

In conclusion, in this report, we propose to use the nucleotide sequence of the velvet-like B gene (*velB*) as a new molecular marker containing a unique microsatellite motif (CAGCAC)_8_ for identification of *M. canis*. The designed, new species-specific primers are capable of distinguishing the target taxon—*M. canis*—from the other dermatophytes, and what is particularly important, the signal does not cross-match with other closely related species of keratinolytic fungi as well as eukaryota, bacteria, viruses, and human DNA confirmed by Primer-BLAST analysis. Moreover, this procedure will be particularly useful in distinguishing zoophilic *M. canis* from anthropophilic *M. audouinii*, which are phylogenetically closely related species and can cause similar clinical manifestations. We hope that the proposed identification assay can be a useful alternative or a supplement to the previously described algorithm of *M. canis* detection [3, 4].
